# Nanoimprinting Solution-Derived Barium Titanate for Electro-Optic Metasurfaces

**DOI:** 10.1021/acs.nanolett.4c00711

**Published:** 2024-04-24

**Authors:** Helena C. Weigand, Ülle-Linda Talts, Anna-Lydia Vieli, Viola V. Vogler-Neuling, Alfonso Nardi, Rachel Grange

**Affiliations:** † ETH Zurich, Department of Physics, Institute for Quantum Electronics, Optical Nanomaterial Group, Auguste-Piccard-Hof 1, 8093 Zurich, Switzerland; ‡ University of Fribourg, Adolphe Merkle Institute, Soft Matter Physics Group, Chemin des Verdiers 4, 1700 Fribourg, Switzerland

**Keywords:** barium titanate, metasurface, tunable, electro-optic, nanoimprint

## Abstract

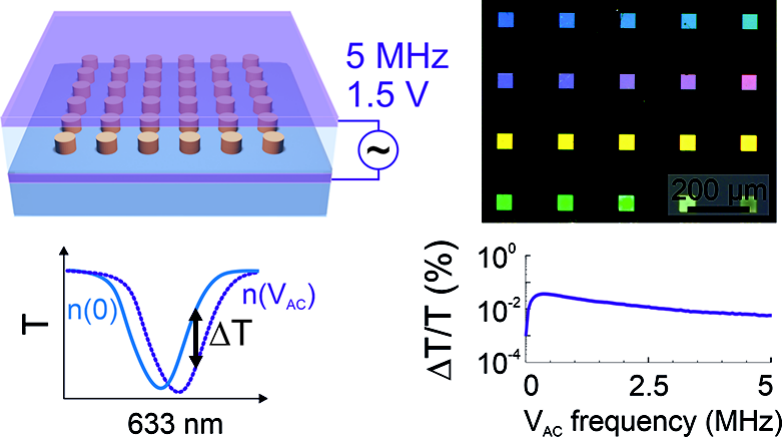

Electro-optic metasurfaces have demonstrated significant potential in enhancing the modulation speed and efficiency for fast and large-scale free-space optical devices. Barium titanate has a strong electro-optic Pockels coefficient, but its availability in thin-film form is restricted due to costly growth processes or low thickness. Here, we fabricated active metasurfaces using an etch-free bottom-up process with sol–gel-based polycrystalline barium titanate with a large electro-optic coefficient similar to bulk lithium niobate. We achieve strong hybrid Mie/surface lattice resonances with a quality-factor of 200 at 633 nm wavelength, enhancing the light–matter interaction and therefore the Pockels effect. The metasurface transmission is electro-optically modulated with up to 5 MHz driving frequency at low voltages of less than 1 V thanks to resonant enhancement of the modulation amplitude by 2 orders of magnitude. This successful demonstration of electro-optic modulation in nanoimprinted barium titanate structures paves the way for low-cost and large-scale free-space modulators or tunable metalenses.

Barium titanate (BTO), with the chemical formula BaTiO_3_, is a perovskite that has been extensively studied for its high dielectric permittivity. This property has enabled the miniaturization of ceramic capacitors in modern electronics.^1^ Additionally, BTO has sparked interest in photonics due to its favorable optical properties, including low-loss transparency in the visible and near-infrared spectral range,^2^ a high refractive index (2.4 at 633 nm),^3^ and a high optical damage threshold. Moreover, the crystal structure of BTO is non-centrosymmetric, resulting in birefringence and nonlinear optical properties such as second harmonic generation (SHG) with a maximal χ^(2)^ of 17 pm/V, which is on the same order as the common nonlinear material lithium niobate.^4^ The tetragonal crystal structure of BTO, with a small but influential difference in lattice constants, also leads to its high electro-optic coefficient of up to 1300 pm/V for bulk BTO, which is the largest Pockels coefficient reported to date.^5^ Epitaxially grown BTO thin-films have shown reduced electro-optic coefficients with *r*_eff_ = 148 pm/V, which still exceeds the widely used thin-film lithium niobate by a factor of 5.^6,7^ Plasmonic electro-optic modulators with modulation speeds in the tens of GHz range were demonstrated by integrating BTO thin-films with silicon photonics.^8^ Dielectric photonic crystal waveguides made of silicon nitride on top of a BTO film have been used to create miniaturized devices with high modulation bandwidth, thanks to the photonic band gap arising from the nanostructures.^9^ However, enhancing modulation efficiencies with resonant nanostructures in BTO itself has been difficult due to the limited thickness and chemical inertness of the metal oxide. Although thin-film BTO has primarily been used for hybrid photonic integrated circuits, it also holds significant potential for metastructure-based flat optics.^10^

Metastructures are periodic arrangements of nanostructures, which are used for phase modulation (e.g., metalenses) or to enhance light–matter interactions (e.g., resonant metasurfaces). The out-of-plane probing avoids coupling losses present in waveguide systems,^11^ while the short light–matter interaction perpendicular to the thin-films can be compensated by resonant designs. BTO metasurfaces have been investigated both theoretically and experimentally for numerous applications such as electro-optic metalenses^12−14^ or hybrid dielectric nanopillar arrays for SHG enhancement.^15^ Research on BTO metasurfaces is, however, not as advanced as that for other nonlinear materials like lithium niobate due to limited availability of thin films. Therefore, alternative approaches using nanoparticles and sol–gel synthesis techniques provide a valuable platform for investigating nanostructures in BTO.^16^

The combination of plasmonic gratings and BTO nanoparticles has been shown to enable enhanced electro-optic modulation. However, these devices suffer from optical losses caused by the presence of gold electrodes. Furthermore, the limited packing density of the nanoparticles significantly reduces the effective field inside the active material.^17^

Solution-based BTO materials are compatible with cost-efficient and large-scale nanofabrication techniques, such as soft-nanoimprint lithography (SNIL),^18^ which have been successfully implemented to produce nonlinear metasurfaces.^19,20^ However, restrictions in nanoparticle packing density limit the refractive index contrast needed for efficient optical nanostructures. Sol–gel derived barium titanate, with higher filling fractions of the imprint mold and thus lower structures’ porosity, is more suitable for high-quality nanoscale photonic applications than nanoparticles. Despite its polycrystalline structure, it outperforms bulk lithium niobate in its effective electro-optic coefficient.^21^ Recently, it was also demonstrated that nanoimprinted sol–gel BTO metasurfaces can resonantly enhance the SHG conversion efficiency in the near-infrared.^22^ Similarly, resonances in metasurfaces can be exploited to enhance electro-optic modulation. This was previously shown in other platforms, such as plasmonic systems integrated with either electro-optic polymers or lithium niobate, or in solely dielectric lithium niobate metasurfaces.^23−25^ While plasmonic structures often rely on surface lattice resonances (SLR),^26^ they are increasingly also implemented in dielectric metasurfaces.^27^ There, Mie resonances of the unit cells couple to Rayleigh anomalies and offer increased quality factors (*Q*-factors), which result in a stronger light–matter interaction.

Here, we demonstrate enhanced electro-optic modulation in sol–gel BTO metasurfaces fabricated via a bottom-up process that does not require any top-down etching steps. The large transparency window of BTO enables modulation of the transmission signal in the visible, unlike most Pockels-effect-based planar devices that operate in the telecom range. Taking advantage of the instant response of the Pockels effect, we show modulation of the metasurface transmission with up to 5 MHz modulation speed. Our fabrication technique allows the integration of metasurfaces with a transparent sandwich ITO electrode configuration, enabling large-scale planar modulators with applied modulation voltages as low as 1.5 V. This work presents the first demonstration of electro-optic modulation in sol–gel derived BTO nanostructures, showing considerable potential for low-cost and large-area active flat photonic devices.

The structures are fabricated on a fused quartz substrate with a transparent layer of 20 nm thick ITO deposited via high vacuum sputtering, which is capped with an electron-beam-deposited 20 nm SiO_*x*_ buffer layer to ensure back-electrode stability. The sol–gel BTO was synthesized based on a previous protocol^21^ and spin-coated on the substrate. Subsequently, it is imprinted by nanostructured polydimethylsiloxane (PDMS), which is described in detail by Talts et al.^22^ and shown in Figure 1a. After removing the PDMS stamp, the BTO metasurface is annealed at 700 °C for 7 h to form a dense polycrystalline structure. XRD and Raman analysis can be found in previous publications.^22,28^ Next, the nanostructures are planarized by spin-coating and soft-baking hydrogen silesquioxane (HSQ), forming a 500 nm thick capping SiO_2_ layer followed by a thin (20 nm) AlO_*x*_ insulation layer. As a final step, the top ITO electrode (20 nm) was sputtered on a selected area on top of the resonant metasurfaces by using a photolithography lift-off process. The metasurface unit cells consist of cylindrical pillars, and their radius as well as the periodicity of the array influence the resonance position, as visible in Figure 1b, where the metasurface period increases from bottom to top and the pillar radius increases from left to right. We can sweep the full color range by varying the radii between 50 and 200 nm and the period from 400 to 800 nm. The distinct colors indicate a sharp resonance that depends on the unit cell and lattice parameters. Figure 1c shows a scanning electron microscopy (SEM) image of an exemplary metasurface before planarization. The SNIL process enables arbitrary scaling of the metasurface size beyond the 50 μm lateral distance demonstrated here. The zoomed-in image of the tilted unit cell in Figure 1d indicates that the pillars are approximately 250 nm in height. This corresponds to a 50% shrinkage from the SNIL master mold, which is compensated for in the design to yield aspect ratios of 2. A cross-section of a similar structure (with a higher residual BTO layer) was obtained by focused ion beam milling. The false-colored SEM in Figure 1e clearly shows successful planarization and reduced porosity compared to nanoparticle BTO structures.^17^ This flexible fabrication method allows for the upscaling of polycrystalline barium titanate metasurfaces with engineered resonances throughout the entire visible to near-IR range.

**Figure 1:**
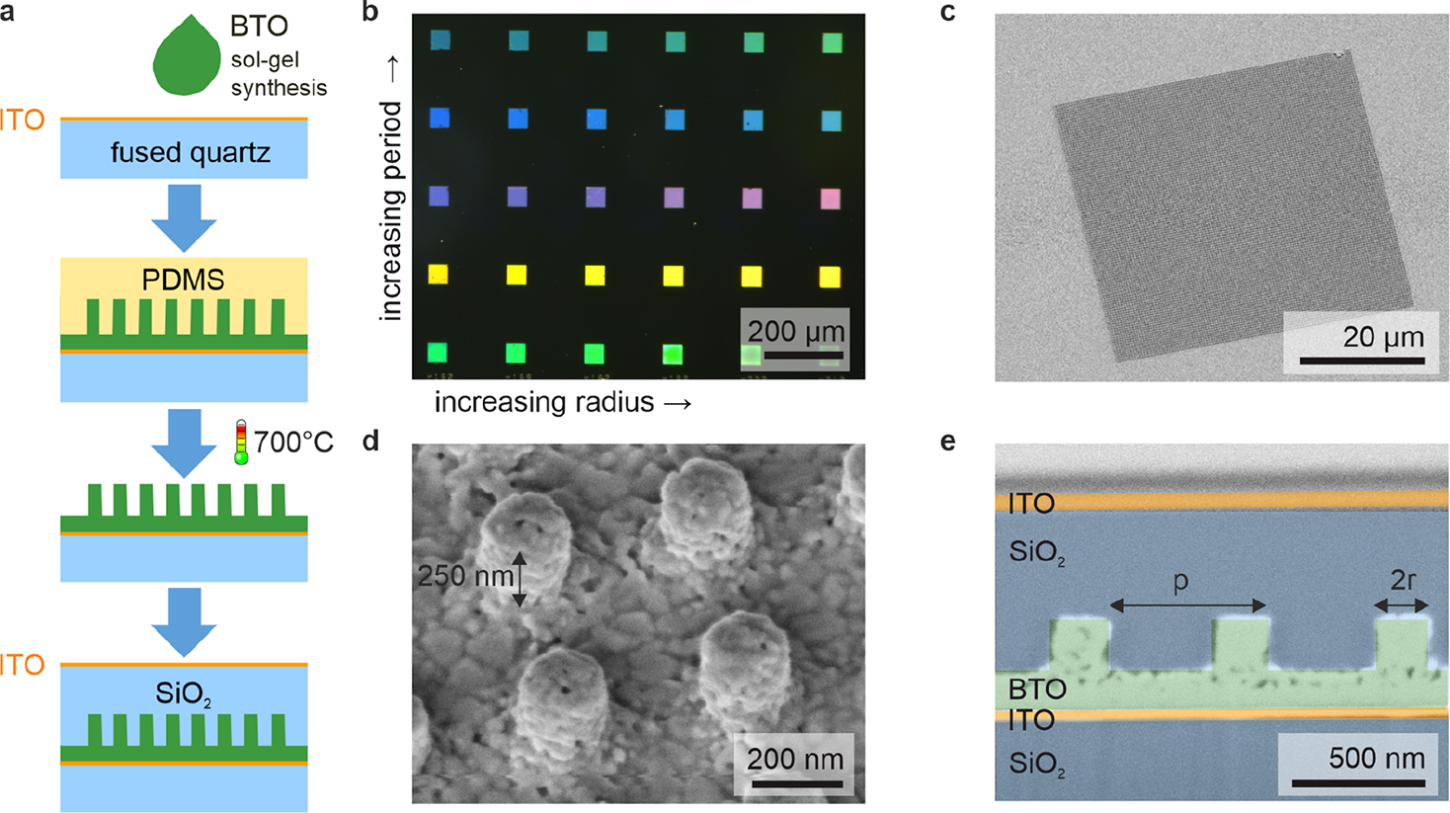
Barium titanate metasurfaces. (a) Schematic of the soft-nanoimprint lithography process and encapsulation of the BTO metasurface. (b) Dark field microscopy of metasurfaces with periodicities from 400 to 800 nm in steps of 100 nm and radii of the unit cells from 80 to 105 nm in steps of 5 nm. (c) Scanning electron microscopy (SEM) image of the imprinted metasurface before encapsulation at 30° tilt. (d) SEM of individual pillar shaped unit cells at 30° tilt. (e) SEM of a cross-section of the embedded structure with false colors.

The nanostructures were investigated in transmission measurements using a supercontinuum laser (NKT SuperK) as a broadband source. The light was impinging on the sample at normal incidence with polarization along the metasurface axis. The transmitted light was collected by a 50× objective (NA 0.55, Zeiss) and fiber-coupled to a spectrometer (Andor SR303i) to acquire the transmission spectra. Figure 2a shows exemplary spectra of metasurfaces with a radius of 75 nm and varying periodicity of 400 to 440 nm (see SI Figure S2c for the full spectral range in the visible). Analyzing the metasurface with period 420 nm reveals a strong transmission dip near 632 nm with a *Q*-factor ( with Δλ as full width at half-maximum of the resonant wavelength *λ*_res_) of 200, which is strong for embedded dielectric metasurfaces with a refractive index contrast of approximately 0.5. As a comparison, state-of-the-art high-*Q* silicon metasurfaces show *Q*-factors of 200–500, while their refractive index contrast is as high as 1.5.^29,30^ The resonant features in Figure 2a exhibit a clear red-shift with increasing periodicity, which is already visible in Figure 1b. Notably, the resonances maintain a high *Q*-factor also for broader periodicity sweeps (SI Figure S2d). An additional dependence on the radius of the unit cell of our metasurface (SI Figure S2a and b for spectra) suggests the formation of Mie-like resonances. Considering the dependence on periodicity, the resonances are likely to originate from a hybridized Mie/surface lattice resonance (SLR).^31^

**Figure 2:**
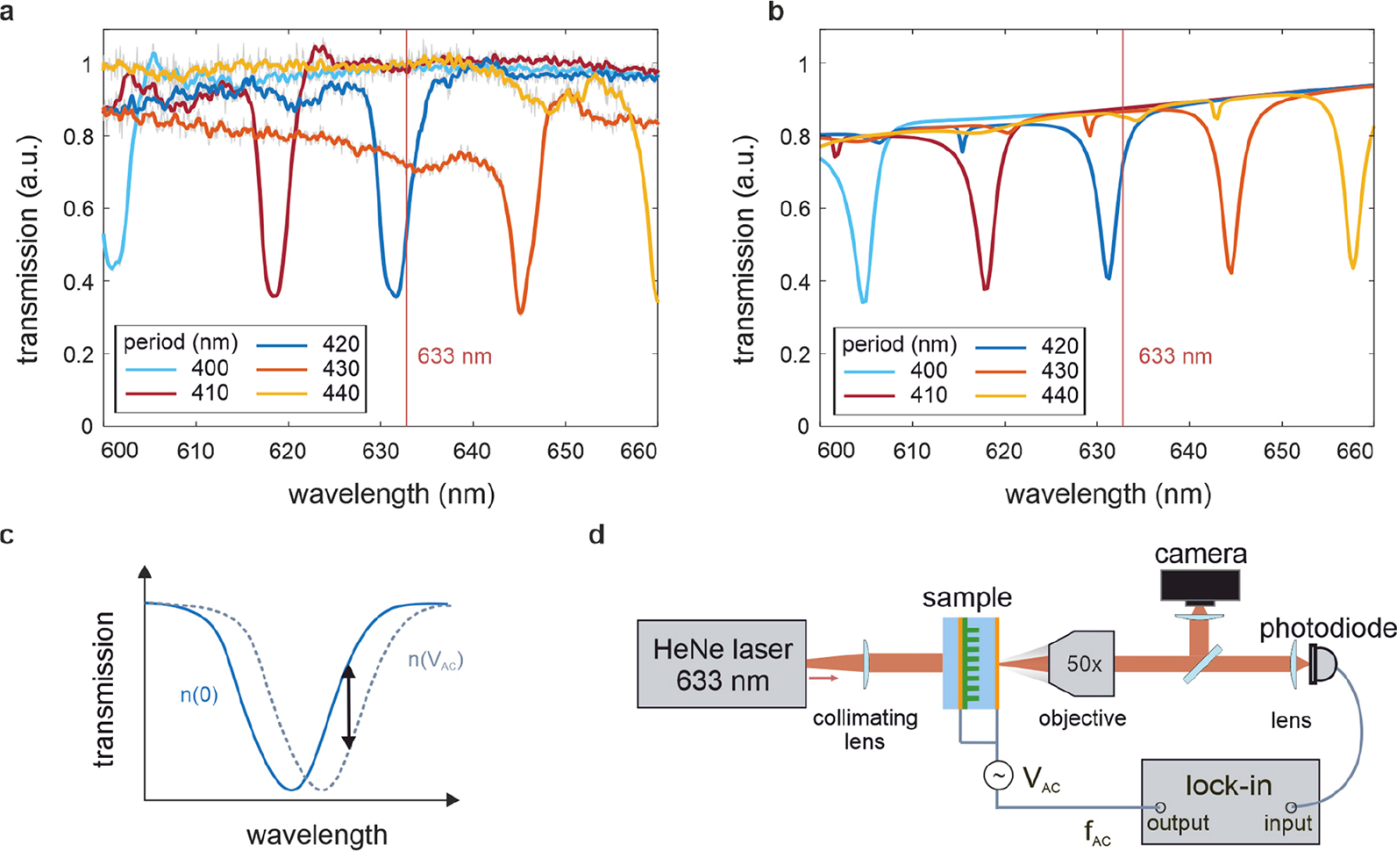
Resonance in BTO metasurface transmission. (a) Measured spectra for a pillar radius of 75 nm. Increasing periodicity of the metasurface redshifts the resonance. (b) FEM simulations of the resonant behavior reveal a hybrid Mie/surface lattice resonance as the origin of the dip in transmission. (c) Principle of electro-optic modulation: the induced change in refractive index will shift the resonance. Probing the spectrum at one wavelength will result in a modulated transmission intensity upon voltage modulation on the sample. (d) Electro-optic modulation is investigated at 633 nm with a polarized laser under normal incidence. The transmitted light is divided by a beamsplitter. One part serves as an imaging path to a camera to define the location on the sample, while the other part of the signal is recorded by a photodiode. This signal is fed into a lock-in amplifier, which simultaneously serves as a driver for the AC voltage applied across the sample.

The experimental results were confirmed by simulations using both the finite difference time domain (FDTD) and finite element methods (FEM). The simulations reproduced a clear signature of an SLR in Figure 2b, where the Rayleigh-anomaly occurs at , with *p* as the lattice constant (420 nm), *n* as the refractive index of the surrounding (*n*_SiO_2__ = 1.4), and *i* as the diffraction order.^32^ The sin(θ) adapts the equation to the incident angle θ, which is normal to the metasurface in our case. This lattice mode hybridizes with the Mie resonances of the individual unit cells, forming sharp Fano-shaped resonances. These resonances shift with the geometrical parameters *r* and *p*, leading to the high theoretical and experimental *Q*-factors observed.^20^ The simulations confirm not only the origin of the resonance but also fully reproduce the measured spectrum.

To investigate the electro-optic effect in the nanoimprinted BTO, we exploit the resonance’s dependence on the refractive index (Figure 2c). The electro-optic Pockels effect describes a linear relation between an externally applied electric field *E* and the refractive index change Δ*n* in the material with applied voltage, following the simplified equation . Here, *r*_eff_ describes the effective electro-optic coefficient of our polycrystalline BTO. The random orientation of the crystalline domains results in a loss of directionality originally given by the material-specific tensors; thus, the crystalline-axis dependent tensor can be replaced by a single coefficient. We investigate the electro-optic effect by choosing a probing wavelength at the steepest slope of the metasurface resonance and observing a change in transmission upon application of a voltage to our sample.^25^Figure 2d shows a sketch of the experimental setup used for electro-optic modulation, with a polarized, continuous wave HeNe laser at 632.8 nm to probe the metasurface. The transmitted light is collected with a 50× objective and divided at a beamsplitter for imaging the sample at a camera and simultaneously collecting the transmission signal with a photodiode. The photodiode signal is fed into a lock-in amplifier, which simultaneously drives an AC voltage on the sample electrodes for the refractive index change (to apply the *E*-field) and locks the incoming optical signal to the AC voltage driving frequency *f*_AC_. This detection system enables the observation of small modulations Δ*T* in transmission, which is expected from the electro-optic effect due to the small refractive index change and the short interaction length compared to integrated circuits. The maximum applied voltage is 1.5 V across the whole sample. However, the majority of this falls over the planarization glass layer, with only approximately 0.03 V applied to the BTO itself. This is due to the significant difference in permittivities between the glass and the perovskite^33^ (SI section 3).

For a modulation frequency of 400 kHz, the experimentally observed modulation is 0.04% (relative to the absolute transmission). This relative modulation was calculated as a ratio , where the change in the transmission signal Δ*T* (read from the lock-in amplifier) is divided by the absolute transmission *T* (read from the photodiode). Assuming an electro-optic coefficient of 27 pm/V^21^ and a refractive index of 1.94 (SI Figure S1, fitted from ellipsometry measurements), we calculate an average expected refractive index shift of approximately Δ*n* = 1.07 × 10^–5^. To confirm the linear electric field dependence of the Pockels modulation, we experimentally sweep the applied voltage (respectively, the electric field *E*) and record the modulation amplitude. As shown in Figure 3a, the relation confirms the Pockels effect as the main driver of the modulation, assuming a linear transmission in the investigated spectral range (SI Figure S3c). The modulation in this device drops by 3 dB at 2 MHz (Figure 3b), while being observed until approximately 5 MHz, where an electrical resonance in the detection circuit starts shielding the electro-optically modulated signal (SI Figure S4e). The low-frequency maximum is a result of the electronic behavior of the sample stack, acting as a high-pass filter for low frequencies and as a low-pass filter for higher values. The physical limitation of the modulation frequency however is not the Pockels effect, which can go up to hundreds of GHz for BTO.^34,35^ Instead, it is the response of the electric circuit of this device itself that limits the bandwidth (mostly due to the resistance of the ITO electrodes and the capacitance of the SiO_2_ layer, derivation in SI section 4). Lowering the resistance of the electrodes or reducing the capacitor’s surface area could increase the bandwidth.^24^ Nevertheless, this result is already significantly faster than, e.g., phase-change- or liquid-crystal-based electric modulation, which is typically limited to low kHz frequencies.^36^

**Figure 3:**
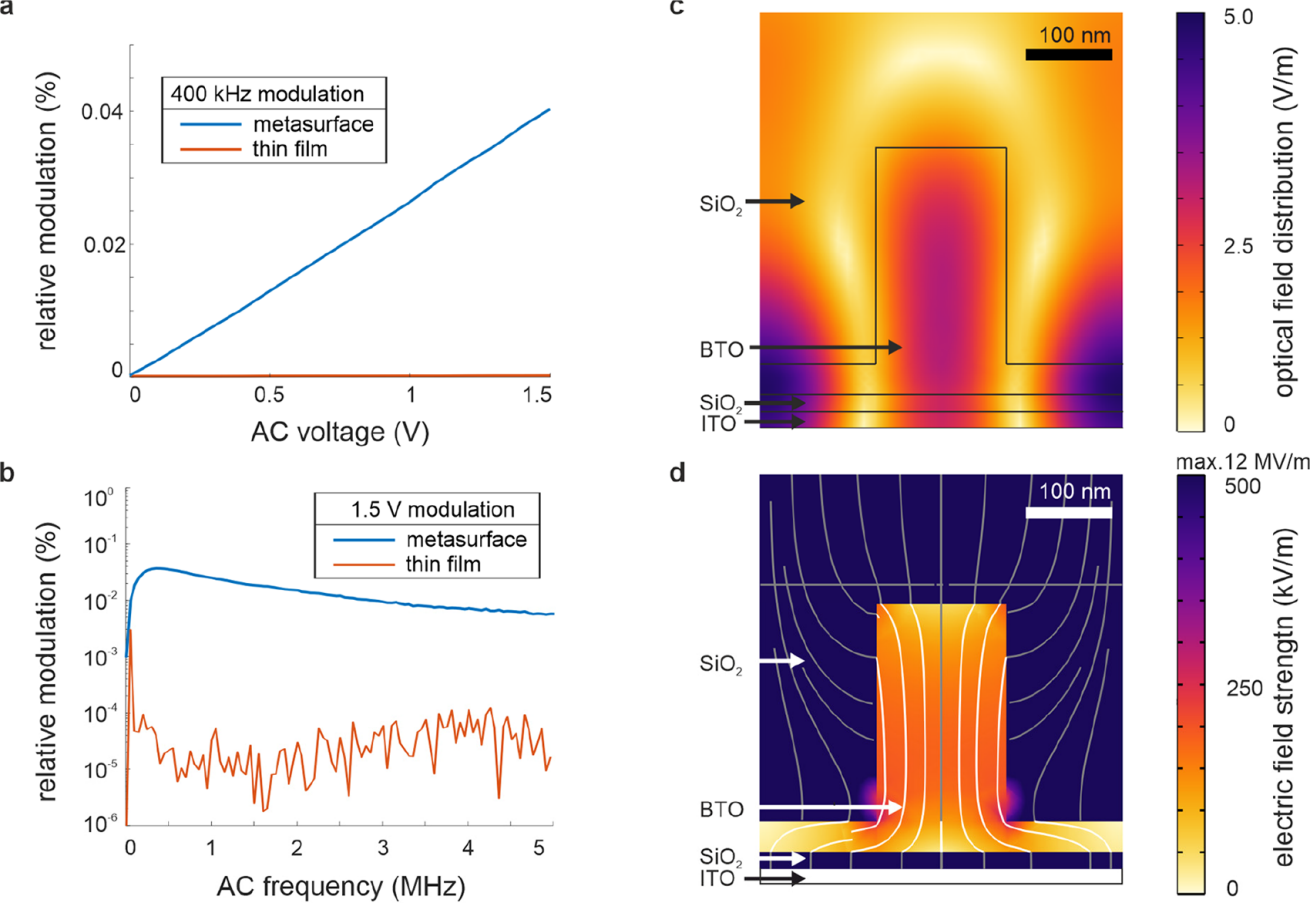
Electro-optic modulation properties. (a) Linear dependence of optical modulation amplitude on driving voltage with a frequency of 400 kHz. (b) Frequency dependence of optical modulation amplitude on driving AC frequency with a voltage of 1.5 V. Note the logarithmic scale. (c) Optical field strength at the probed wavelength (632.8 nm). (d) Electric field distribution from applied voltage (colorbar saturated at 500 kV/m, max. field strength 12 MV/m in SiO_2_).

While an unstructured BTO layer of equal thickness as the pillar height yields a 400 kHz-modulation amplitude of 6 × 10^–5^%, which is barely above the detection level, the resonance of the metasurface increases the modulation to 0.04%, which is a factor of 600 improvement. In this device, the polycrystalline BTO metasurface modulation is not affected by DC biasing up to 30 V applied over the whole device structure. It is known from the literature that thin BTO sol–gel films have high coercive fields, and it is likely that too low electric fields were applied to the nanostructures to successfully orient their crystalline domains.^37^ The influence of the metasurface on the modulation depends on the *Q*-factor of the resonance in transmission, as shown in spectrally probed modulated metasurfaces.^25^ There, the strongest modulation is observed at the maximum of the transmission derivative, which is the steepest position of the resonance curve. The strength of electro-optic modulation is also affected by the spatial overlap between the electrical field distribution and the optical field distribution. We investigate this by FEM simulations of the DC field applied top-down via the ITO electrodes to retrieve the spatially resolved refractive index change in the BTO. This Δ*n* map is subsequently used for FEM simulations to model the influence of the electric field on the optical resonance and its electromagnetic field distribution. These simulations predict a transmission modulation of 0.02%, which closely matches the experimental results (detailed derivation in SI section 3). Figure 3c shows the optical field distribution in the resonant metasurface at the probe wavelength of 633 nm. The optical field is homogeneously localized in the center of the BTO pillar structure as well as in the small residual layer (35 nm). Figure 3d, instead, shows the distribution of the electric field and thus refractive index change in the sandwich electrode structure (note the colorbar is capped at 500 kV/m for readability). While the SiO_2_ planarization layer on top of the metasurfaces retains most of the electric field due to its much smaller relative permittivity, the BTO nanostructure concentrates the electric field inside the pillar. This results in a strong field overlap in the center of the BTO pillar for the electric and optical fields, allowing efficient refractive index change induced modulation of the transmission for our device.^38^

While our structure employs high local control over the two fields interacting via the Pockels effect in the material, the main limitation of our device is the electric field drop over the SiO_2_. This could be addressed by reducing the thickness of this planarization layer but would simultaneously result in higher optical losses due to the proximity of plasmonic materials to the optical resonance. Additional engineering would be required to find an optimal trade-off, although the high electric field drop in the surrounding material of BTO thin-film modulators remains a known limitation of these devices.^35^ A potential solution is the use of planarization layer materials with higher relative permittivity such as TiO_2_ instead of glass-based materials.

In this work, we electro-optically modulated a polycrystalline sol–gel BTO metasurface, which we fabricated using highly scalable bottom-up soft-nanoimprint lithography. The designed hybridized Mie/surface lattice resonance around 633 nm wavelength enhances the Pockels effect in BTO, which is utilized for modulating the metasurface transmission. This is the first demonstration of the electro-optic effect in a nanostructured sol–gel, which lays the foundation for low-cost and large-scale fabrication of tunable planar modulators or flat optics based on polycrystalline BTO. We show that a nanostructure induced resonance results in a 600-fold increase in modulation of the metasurface compared to a flat BTO sol–gel film. This highlights the importance of enhanced light–matter interaction and overlap engineering of optical and electrical field distributions. The position of the resonance and thus the modulator wavelength can be tuned with the geometrical parameters of the metasurface over a broad visible to near-infrared range while maintaining high *Q*-factors of 200. The modulation upon application of an AC voltage shows a linear dependence on the voltage amplitude, indicating the Pockels effect as the source of modulation. We report modulation frequencies up to 5 MHz, limited by low-pass filtering of the device equivalent electrical circuit rather than the Pockels effect in BTO, which has been measured to exceed GHz frequencies. The demonstrated modulation is considerably faster than current spatial light modulators such as those based on liquid crystals. Despite the sol–gel BTO having a smaller effective electro-optic coefficient and thus modulation strength than bulk BTO, voltages of less than 0.1 V are sufficient to drive the modulation of our metasurface transmission. This demonstrates the significant potential of bottom-up sol–gel-based fabrication, which allows for inexpensive and scalable modulators with low power consumption. Furthermore, metalenses made via soft-nanoimprinted BTO^39^ have demonstrated critical dimensions and aspect ratios that are not feasible using top-down fabrication. Our demonstration of electro-optic modulation in the visible spectral range provides an experimental platform to implement the many numerical studies proposing electro-optic modulation of BTO-based metalenses.^12−14^ The combination of SNIL with the push to improve the electro-optic coefficient of sol–gel derived BTO^40^ is expected to pave the way for high-speed modulators as well as electrically tunable metalenses. Lastly, the polycrystallinity of solution-based BTO structures opens the way to the field of randomness-based and thus broadband nonlinear phenomena.^41^

## Supporting Information Available

Details of simulations, radius dependence of resonance, full transmission spectra, simulation of transmission shift, and electrical circuit characterization (PDF)
