# Supplementary material for: Nanoimprinting Solution-Derived Barium Titanate for Electro-Optic Metasurfaces
Source: Nano Lett. 2024 Apr 24;24(18):5536–42. doi: 10.1021/acs.nanolett.4c00711 (PMC11082927; doi:10.1021/acs.nanolett.4c00711)
Supplement: Supplementary file 1 — nl4c00711_si_001.pdf [file nl4c00711_si_001.pdf]

Supplementary Information

Nanoimprinting solution-derived barium titanate for electro-optic metasurfaces

Helena C. Weigand[1], Ülle-Linda Talts[1], Anna-Lydia Vieli[1], Viola V. Vogler-Neuling[1,2], Alfonso Nardi[1], Rachel Grange[1]*

[*] Corresponding Author: hweigand@phys.ethz.ch

[1] ETH Zurich, Department of Physics, Institute for Quantum Electronics, Optical Nanomaterial Group, Auguste-Piccard-Hof 1, 8093 Zurich, Switzerland

[2] University of Fribourg, Adolphe Merkle Institute, Soft Matter Physics Group, Chemin des Verdiers 4, 1700 Fribourg, Switzerland

Content

1. Refractive index of BTO 2
2. Transmission spectra 3
3. Simulation of transmission modulation 4
4. Electrical circuit calculations 5

1. Refractive index of BTO

The refractive index of the annealed BTO sol-gel is determined via ellipsometry measurements (Woollam VASE) and fitted to the experimental data. The refractive index is an effective average over all grains and impurities included in the annealed BTO layer. The dispersive behavior shown in Figure S1 was used for the FDTD and FEM simulations and is derived from these optical measurements.

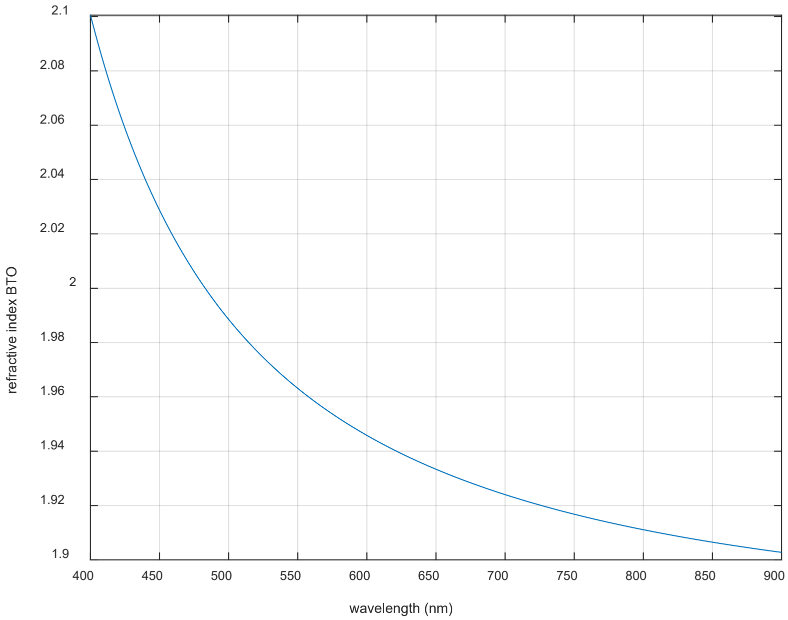

Figure S1: Real part of the refractive index of BTO as fitted from ellipsometry measurements and used for FEM and FDTD simulations, with a refractive index of 1.94 at the probed wavelength of 633 nm.

2. Transmission spectra – dependence on radius

The metasurface transmission can be varied by the geometrical parameters. Besides the periodicity (discussed in the main text), also the radius of the unit cell pillars shifts the resonance. Figure S2a shows experimentally retrieved spectra (setup described in the main text) next to FEM simulations on BTO metasurfaces with a periodicity of 420 nm (Figure S2b). A redshift of the resonance position with increasing pillar radius is clearly visible and hints towards the influence of Mie-like resonances on the transmission. In addition to that, the signature of an SLR as mentioned in the main text are also apparent (blue shifted from the main resonance dip), both in the simulation and the measurement.

Figure S2c shows the full visible wavelength range of the experimentally retrieved transmission for metasurfaces with periodicity 400 nm. Instead of normalizing to an unstructured BTO thin-film, this time the spectra are referenced to a quartz substrate to illustrate the Fabry-Perot behavior visible in the spectrum. The black line shows the undistorted thin-film Fabry-Perot pattern used for reference in the other transmission plots given. Figure S2d shows the transmission spectrum for a periodicity of 500 nm and while the resonances redshift compared to S2c (as expected from Figure 2a in the main text), the high Q-factor of the resonances is preserved also for different resonance positions. Interestingly, for the higher periodicity already higher orders of the resonance appear at lower wavelengths.

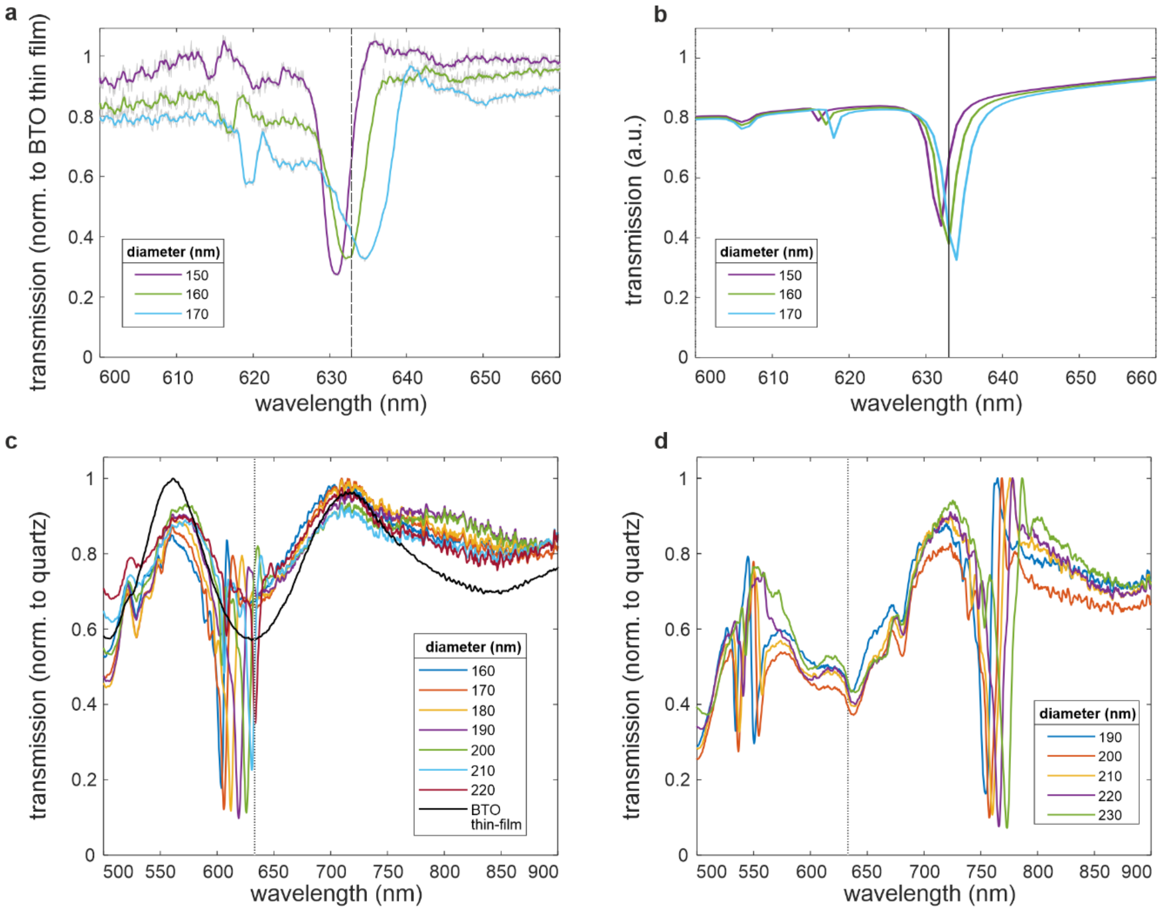

Figure S2: Transmission spectra of BTO metasurface of 420 nm periodicity with varying radii. a) Experimentally retrieved spectra. b) FEM simulations of transmission (influence of Fabry-Perot effect of SiO_2 layer visible in broad modulation of curve). c) Full spectrum of the acquired transmission curve for metasurfaces with periodicity 400 nm. d) Full spectrum for metasurfaces with periodicity 500 nm, which show a redshift of the resonance position while preserving high Q-factors.

3. Simulation of transmission modulation

In order to model the change of transmission in our system, in a first step the change of refractive index needs to be determined. As seen in the main text, the Pockels effect is defined as $\Delta n = \frac{1}{2} \cdot r_{eff} \cdot n^3 \cdot E$. While the refractive index is determined from ellipsometry to be 1.94 at 633 nm, the effective Pockels coefficient is taken from literature to be 27pm/V [R1]. The electric field E is determined from electrostatic FEM simulations (Figure 3d main text), where a voltage of 1.5V is applied to one ITO electrode. The simulation assumes a relative permittivity of the SiO_2 layers of ε_r=3.9 and a BTO relative permittivity of ε_r=500. Note that, in order not to underestimate the electric field on the BTO, we considered a relative permittivity lower than that reported in literature (ε_r=1000) to account for reduced domain size and voids in our sol-gel structures. [R2] The simulation results in an average electric field of 167kV/m in the BTO pillar (see main text, Fig.3d). Our refractive index change upon application of 1.5 V will be on average $\Delta n = 1.07 \cdot 10^{-5}$, while the spatial distribution of the electric field given in Figure 3d in the main text translates on the spatial distribution of the local refractive index change. Figure S3 shows FEM simulated transmission spectra for the investigated BTO metasurface for no applied voltage, a voltage of 1.5 V and an extreme case of 150 V ($\Delta n = 1.07 \cdot 10^{-3}$).

While the experimental results yield a modulation of 0.04 %, the FEM simulations predict a 0.02 % transmission change for a modulation with 1.5 V ($\Delta n = 1.07 \cdot 10^{-5}$) as retrieved from Figure S3c. This good agreement in order of magnitude shows a profound understanding of the transmission and modulation behavior of our device, while the small deviation in absolute values can be explained by slightly different material values than those taken from literature, in particular the electro-optic coefficient and relative permittivity of the BTO.

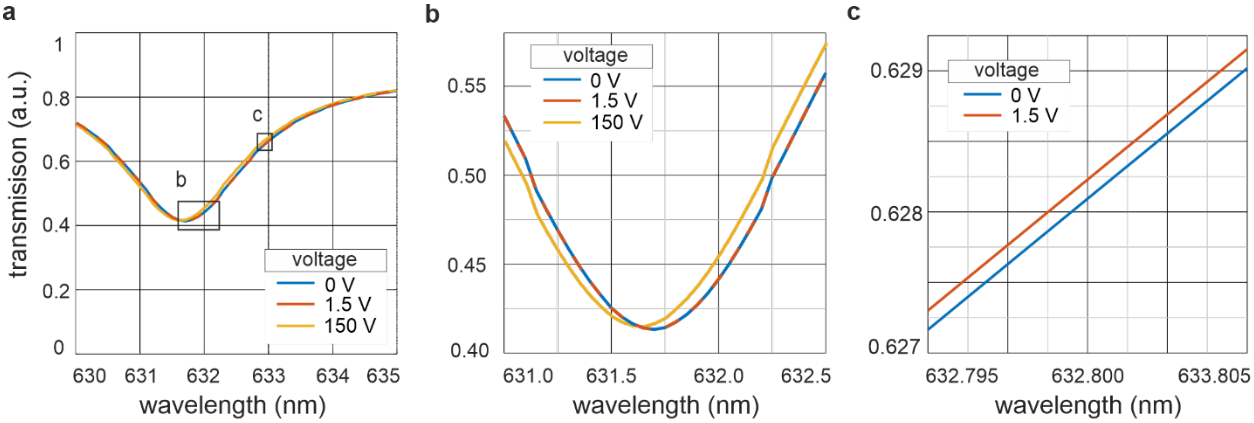

Figure S3: FEM simulation of transmission of the BTO metasurface device upon refractive index change induced by voltage application. a) Full resonance range for exemplary applied electric fields. b) Resonant position zoomed in as indicated in a). The difference in spectra for the expected order-of-magnitude change is too small to be resolved (dashed lines on top of each other). c) Spectrum at resonance slope for the applied voltage of 1.5 V, zoomed in as indicated in a) and showing a transmission change from 66.833% to 66.847 %.

4. Electrical circuit calculations

In this section, we derive the electrical frequency response of a typical device (shown in Fig. S4b).

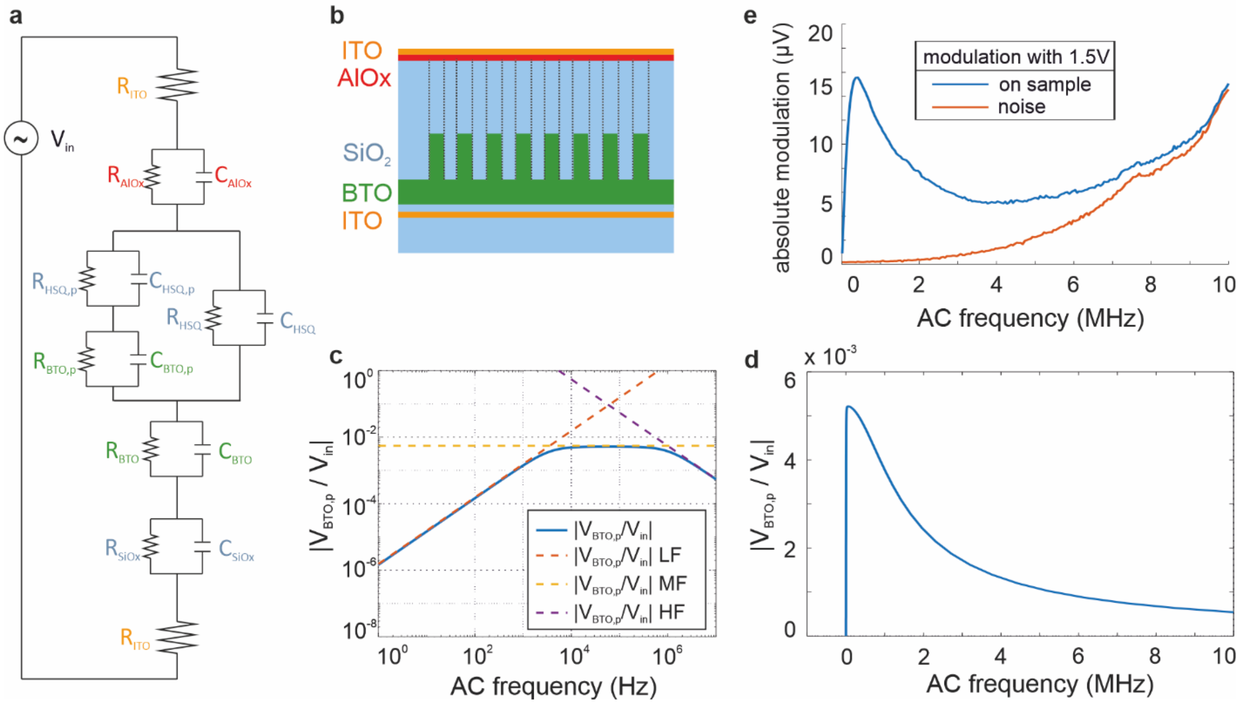

Figure S4: Electrical analysis of the sample stack. a) Schematic of the electrical circuit used for modelling the device. b) Illustration of the sample geometry to clarify the use of parallel circuits for pillars and glass-areas. c) Logarithmic Bode plot with different regimes of the frequency behavior of the circuit. d) Linear Bode plot which shows the peak-like behavior of the field drop over the pillars for low frequencies (with a maximum at 75 kHz AC frequency). e) Experimentally retrieved frequency dependence of the optical modulation. Comparing to d), the low frequency peak clearly stems from the electric behavior of the sample itself. The increase at high frequencies (> 7 MHz) is a spurious signal from the lock-in detection scheme, as its also present in the reference measurement without transmitted laser beam. We chose 5 MHz as a cut-off, because there the absolute value of the spurious signal rises to more than 50% of the magnitude of the absolute sample-modulation signal.

To this aim, we model each layer of different material as a separate electronic component, consisting of a capacitor and a resistor in parallel. The resulting electronic circuit is depicted in Fig. S4a. Special care is needed for the layer occupied by the spin-on glass HSQ (SiO_x) and the BTO pillars. Here, we make the approximation of considering the field lines perpendicular to the electrodes. This allows us to separate the area of the pillars from the surrounding HSQ and consider them as different electronic components in parallel, as highlighted in Fig. S4b. Moreover, we assume that the pillars are identical, allowing us to condense them in a single component with impedance $Z_{HSQ,p} + Z_p$, characterized by an area A_{pillars} equal to the total one covered by the pillars, in parallel to a device with impedance Z_{HSQ} and area $A_{HSQ} = A_{TOT} - A_{\text{pillars}}$, where A_{TOT} is the area covered by the electrodes.

The total area covered by the pillars is given by

$$A_{\text{pillars}} = N_{\text{MS}} \times N_{\text{pillars}} \times A_{\text{s.p.}}\,,$$

where $N_{\text{MS}} = 36$ is the number of metasurfaces covered by the ITO electrodes, $A_{\text{s.p.}} = \pi(75\text{ nm})^2$ is the area of a single pillar, and N_{pillars} is the number of pillars per metasurface, given by

$$N_{\mathrm{pillars}} = \left(\frac{L_{\mathrm{MS}}}{p}\right)^2 = \left(\frac{50\ \mu\mathrm{m}}{420\ \mathrm{nm}}\right)^2,$$

with L_{MS} the length of a single metasurface, and p the periodicity of the pillars.

We measured the resistance of the ITO layer to be $R_{ITO} \approx 1\ \mathrm{k}\Omega$. For the other layers, we obtained the values of the resistance using the formula

$$R_{\mathrm{x}} = \rho_{\mathrm{x}} \frac{t_{\mathrm{x}}}{A_{\mathrm{x}}},$$

where A_{x} is the area, ρ_{x} is the resistivity and t_{x} is the thickness of the x –th layer, respectively.

The capacitance is estimated using the formula

$$C_{\mathrm{x}} = \frac{A_{\mathrm{x}}\varepsilon_{r,\mathrm{x}}\varepsilon_0}{d_{\mathrm{x}}},$$

where ε_0 is the dielectric permittivity of vacuum, A_{x} is the area, d_{x} is the thickness and $\varepsilon_{r,\mathrm{x}}$ is the relative permittivity of the x –th layer. The values of each parameter and the resulting resistance and capacitance are reported in Table S1.

Given the electronic circuit in Fig. S4a, we calculate the frequency response using the Laplace transform. The voltage drop over the pillars is given by the following expression:

$$V_{\mathrm{BTO,p}} = V_{\mathrm{in}} \frac{Z_{TOT}}{2\ R_{\mathrm{ITO}} + Z_{\mathrm{AlO_x}} + Z_{\mathrm{TOT}} + Z_{\mathrm{BTO}} + Z_{\mathrm{SiO_2}}} \frac{Z_{\mathrm{BTO,p}}}{Z_{\mathrm{BTO,p}} + Z_{\mathrm{HSQ,p}}}$$

where Z_{TOT} is

$$Z_{TOT} = \frac{1}{\frac{1}{Z_{\mathrm{BTO,p}} + Z_{\mathrm{HSQ,p}}} + \frac{1}{Z_{\mathrm{HSQ}}}},$$

while the impedance of the x –th component is

$$Z_{\mathrm{x}} = \frac{R_{\mathrm{x}}}{1 + sC_{\mathrm{x}}R_{\mathrm{x}}}.$$

We report the Bode magnitude plot versus frequency in Fig. S4c and S4d in logarithmic and linear scale, respectively. It provides a qualitative explanation to the low-frequency peak measured in the optical response of the device (Fig. S4e). The discrepancy in the absolute value of the voltage drop compared to the FEM simulations (4 times larger in the simulations) and the lower frequency of the peak of the electrical compared to the optical frequency response can be found in the approximation of field lines perpendicular to the electrodes. From the FEM simulations reported in Fig. 3d in the main text, it is in fact clear that there is a large contribution of the field which is coming from the side of each pillar, that we are not considering in this treatment.

Nevertheless, the proposed circuit highlights the factors that limit the frequency response. At low frequency, the impedance divider is dominated by the high value of the capacitance of the HSQ, while

the BTO pillars are limited by the value of their resistance. Therefore, for low frequency the response is a simple high pass filter of the form:

$$\left.\frac{V_{\mathrm{BTO,p}}}{V_{\mathrm{in}}}\right|_{\mathrm{low\ freq.}} = s\, C_{\mathrm{HSQ,p}}\, R_{\mathrm{BTO,p}}\,.$$

At mid-frequencies, here 10^5 Hz, the impedance of the BTO pillars' capacitor becomes smaller than their resistance, leading to a simple capacitance divider

$$\left.\frac{V_{\mathrm{BTO,p}}}{V_{\mathrm{in}}}\right|_{\mathrm{mid\ freq.}} = \frac{C_{\mathrm{HSQ,p}}}{C_{\mathrm{BTO,p}} + C_{\mathrm{HSQ,p}}}\,.$$

This capacitance divider is what limits the voltage drop over the BTO pillars. Reducing the thickness of the HSQ layer, or resorting to a cladding material with higher electrical permittivity would lead to an increased field inside the BTO pillar.

Finally, at high frequency the impedance of the HSQ becomes smaller than the ITO resistance, leading to a low pass filtering:

$$\left.\frac{V_{\mathrm{BTO,p}}}{V_{\mathrm{in}}}\right|_{\mathrm{high\ freq.}} = \frac{C_{HSQ}}{C_{BTO} + C_{HSQ}}\,\frac{1}{1 + sC_{HSQ}\,2R_{ITO}}\,.$$

Therefore, one would need to reduce the value of the ITO resistance to use the device efficiently at higher frequencies.

Material	$\boldsymbol{A}$	$\boldsymbol{t_x}$	$\boldsymbol{\rho_x}$	$\boldsymbol{\varepsilon_r}$	$\boldsymbol{R_x}$	$\boldsymbol{C_x}$
BTO (residual)	$1.4\ \mathrm{mm}^2$	35 nm	$10^4\ \Omega\ \mathrm{m}$ [R1]	500	$250\ \Omega$	177 nF
AlO_x	$1.4\ \mathrm{mm}^2$	25 nm	$10^{14}\ \Omega\ \mathrm{m}$ [R3]	6.7	$4.3 \times 10^{13}\Omega$	3.3 nF
SiO_2	$1.4\ \mathrm{mm}^2$	20 nm	$10^{15}\ \Omega\ \mathrm{m}$ [R4]	3.9	$1.4 \times 10^{13}\Omega$	2.4 nF
HSQ	$1.39\ \mathrm{mm}^2$	600 nm	$10^{15}\ \Omega\ \mathrm{m}$ [R4]	3.9	$4.3 \times 10^{14}\Omega$	80 pF
BTO, p (pillar)	$0.01\ \mathrm{mm}^2$	250 nm	$10^4\ \Omega\ \mathrm{m}$ [R1]	500	$2.8 \times 10^5\Omega$	160 pF
HSQ, p (pillar)	$0.01\ \mathrm{mm}^2$	350 nm	$10^{15}\ \Omega\ \mathrm{m}$ [R4]	3.9	$3.9 \times 10^{16}\Omega$	0.9 pF

Table S1: The table reports the values of the relevant parameters, as well as the resulting capacitance and resistance for each electronic component of the modeled circuit.

References:

R1. Edmondson, B. I. *et al.* Epitaxial, electro-optically active barium titanate thin films on silicon by chemical solution deposition. *J. Am. Ceram. Soc.* **103**, 1209–1218 (2020).

R2. Panomsuwan, G. & Manuspiya, H. Structural and dielectric properties of sol–gel derived Ba1–xSrxTiO3 (0 ≤ x ≤ 0.5) ceramics for energy storage applications. *Mater. Res. Express* **6**, 026310 (2018).

R3. Groner, M. D., Elam, J. W., Fabreguette, F. H. & George, S. M. Electrical characterization of thin Al2O3 films grown by atomic layer deposition on silicon and various metal substrates. *Thin Solid Films* **413**, 186–197 (2002).

R4. El-Kareh, B. Fundamentals of Semiconductor Processing Technology. *Fundam. Semicond. Process. Technol.* (1995) doi:10.1007/978-1-4615-2209-6.
